# Improving Diastolic and Microvascular Function in Heart Transplantation with Donation after Circulatory Death

**DOI:** 10.3390/ijms241411562

**Published:** 2023-07-17

**Authors:** Lars Saemann, Adrian-Iustin Georgevici, Fabio Hoorn, Nitin Gharpure, Gábor Veres, Sevil Korkmaz-Icöz, Matthias Karck, Andreas Simm, Folker Wenzel, Gábor Szabó

**Affiliations:** 1 Department of Cardiac Surgery, University Hospital Halle (Saale), 06120 Halle (Saale), Germany; 2 Department of Cardiac Surgery, Heidelberg University Hospital, 69120 Heidelberg, Germany; 3 Department of Anaesthesiology, St. Josef Hospital, Ruhr-University Bochum, 44791 Bochum, Germany; 4 Faculty Medical and Life Sciences, Furtwangen University, 78054 Villingen-Schwenningen, Germany

**Keywords:** heart transplantation, microvascular dysfunction, donation after circulatory death, machine perfusion, machine learning, Custodiol-N, HTK-N, Laser-Doppler-Flow, Laser-Doppler-Perfusion, microcirculation

## Abstract

The impact of the machine perfusion of donation after circulatory death (DCD) hearts with the novel Custodiol-N solution on diastolic and coronary microvascular dysfunction is unknown. Porcine DCD-hearts were maintained four hours by perfusion with normothermic blood (DCD-B), hypothermic Custodiol (DCD-C), or Custodiol-N (DCD-CN), followed by one hour of reperfusion with fresh blood, including microvascular and contractile evaluation. In another group (DCD group), one hour of reperfusion, including microvascular and contractile evaluation, was performed without a previous maintenance period (all groups N = 5). We measured diastolic function with a balloon catheter and microvascular perfusion by Laser-Doppler-Technology, resulting in Laser-Doppler-Perfusion (LDP). We performed immunohistochemical staining and gene expression analysis. The developed pressure was improved in DCD-C and DCD-CN. The diastolic pressure decrement (DCD-C: −1093 ± 97 mmHg/s; DCD-CN: −1703 ± 329 mmHg/s; DCD-B: −690 ± 97 mmHg/s; *p* < 0.05) and relative LDP (DCD-CN: 1.42 ± 0.12; DCD-C: 1.11 ± 0.13; DCD-B: 1.22 ± 0.27) were improved only in DCD-CN. In DCD-CN, the expression of eNOS increased, and ICAM and VCAM decreased. Only in DCD-B compared to DCD, the pathways involved in complement and coagulation cascades, focal adhesion, fluid shear stress, and the IL-6 and IL-17 pathways were upregulated. In conclusion, machine perfusion with Custodiol-N improves diastolic and microvascular function and preserves the microvascular endothelium of porcine DCD-hearts.

## 1. Introduction

Diastolic dysfunction in cardiac allografts is a risk factor for mortality [1]. The development of diastolic dysfunction is associated with the presence of coronary microvascular dysfunction (CMD) [2]. Furthermore, CMD has been discussed as a phenotypic manifestation or preceding factor of acute allograft rejection [3]. CMD could also be the main contributor to diastolic dysfunction after heart transplantation. The mechanisms which contribute to the development of CMD are diverse [4]. The coronary microvascular endothelium is a crucial structure that regulates vessel tone, vascular resistance, and microvascular blood flow—three characteristics that are impaired in the presence of CMD [4].

Heart transplantation after donation from circulatory death (DCD) is increasing, and the results are comparable to donation from brain death [5]. Currently, a great deal of research is being published investigating various aspects of DCD heart transplantation in clinical and experimental settings. Nevertheless, the occurrence of CMD has not been investigated in the context of DCD.

Endothelial dysfunction in the coronary microvasculature, a major mechanistical contributor to CMD [6,7], can be initiated by ischemia/reperfusion (I/R) injury. Recently, we could show in an experimental study that continuous machine perfusion (MP) with crystalloid cardioplegic solutions instead of blood perfusion reduces I/R injury in the myocardium of DCD hearts and enhances contractility compared to blood perfusion [8]. Nevertheless, no difference regarding systolic function was visible between traditional Custodiol (histidine-tryptophane-ketoglutarate; HTK) and the novel Custodiol-N solution (HTK-N) cardioplegic preservation solutions. The main differences between Custodiol-N and traditional Custodiol are the supplementation by the intracellular iron chelator LK-614, the extracellular iron chelator deferoxamine, the amino acids alanine and arginine, the increased ketoglutarate concentration, the partial supplementation of histidine by N-α-acetyl histidine, and the reduced chloride concentration [9].

Custodiol-N was developed for preserving the myocardial and vascular function of donor hearts. Consequently, we hypothesize that it is superior to blood and traditional Custodiol to preserve microvascular function during the MP of DCD hearts. Thus, in the present study, we investigated the effect of MP with Custodiol-N on diastolic and microvascular function in porcine DCD hearts. Furthermore, we applied a pathway analysis of gene expression to itemize molecular factors that could play a major role in developing or preventing diastolic and microvascular dysfunction.

## 2. Results

### 2.1. Systolic and Diastolic Function

**Figure 1:**
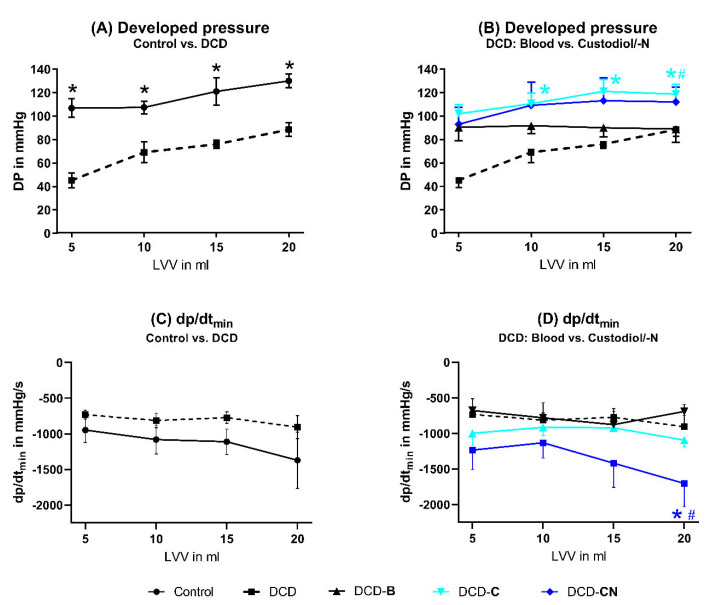
Systolic and diastolic function. * *p* < 0.05 compared to DCD and ^#^ *p* < 0.05 compared to DCD-B. B: Blood. DCD: Donation after circulatory death. C: Custodiol. CN: Custodiol-N. LVV: Left-ventricular volume.

The developed pressure was significantly lower in the DCD hearts compared to the control hearts (Figure 1). Both MP with Custodiol and Custodiol-N improved the developed pressure of DCD hearts and also resulted in superior contractility compared to DCD-B. No difference could be shown between the groups DCD-C and DCD-CN. The diastolic parameter dp/dt_min_ was also impaired in the DCD group compared to the control group. The groups DCD-B and DCD-C did not show a dp/dt_min_ that was different from the DCD group. After Custodiol-N, perfusion dp/dt_min_ was significantly improved compared to the groups DCD and DCD-B.

### 2.2. Coronary Macro- and Microcirculation

**Figure 2:**
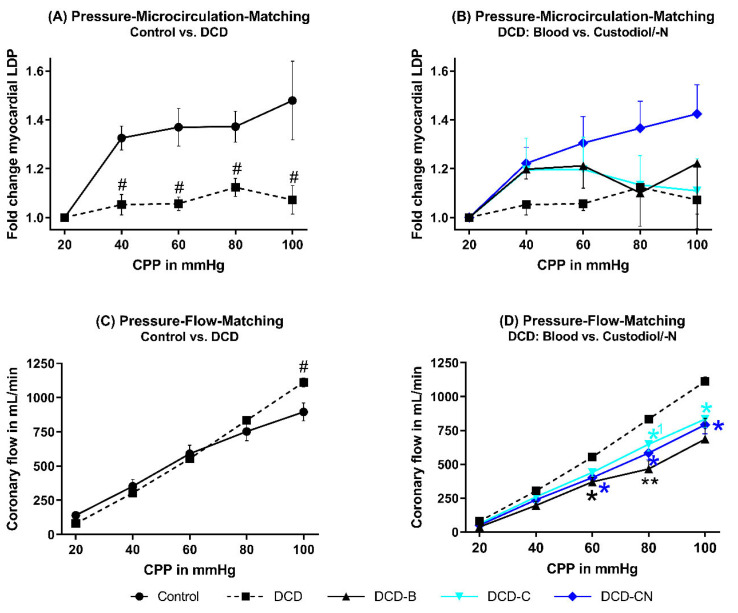
Coronary macro- and microvascular function. (**A**,**B**) Myocardial microcirculation in dependence of different CPP. (**C**,**D**) Total coronary flow in dependence of different CPP. ^#^ *p* < 0.05 compared to Control. * *p* < 0.05, ** *p* < 0.001, and *^1^ *p* = 0.065 compared to DCD. B: Blood. DCD: Donation after circulatory death. CPP: Coronary perfusion pressure. C: Custodiol. CN: Custodiol-N.

**Figure 3:**
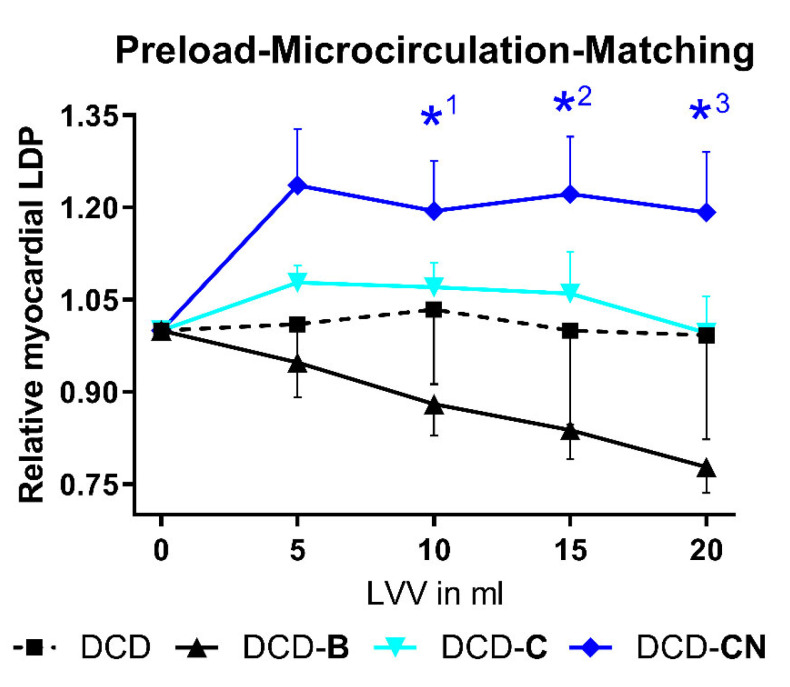
Coronary microvascular function. *^1^ *p* = 0.059, *^2^ *p* = 0.062, and *^3^ *p* = 0.056 compared to DCD-B. DCD: Donation after circulatory death. C: Custodiol. CN: Custodiol-N. B: Blood. LVV: Left-ventricular volume.

An increase in CPP resulted in a majorly improved LDP in the DCD-CN group, slightly improved LDP in the DCD-C and DCD-B groups, and only minor changes in the DCD group (Figure 2B). In the groups DCD-C and DCD-CN, a rise in CPP from 20 to 40 mmHg resulted in a comparable increase in LDP. Nevertheless, a further rise in CPP above 40 mmHg only improved LDP in the DCD-CN group, while in the DCD-C group, LDP remained unchanged. The total coronary flow showed a steep, linear relationship to CPP in DCD hearts (Figure 2D). In the groups DCD-B, -C, and -CN, the flow curve showed a slight infliction by an increased CPP, but all the flow curves of these groups showed a similar course. In groups DCD and DCD-C, RLDP was not majorly affected by an LV filling. In the DCD-B group, LDP decreased by a rising LV filling (Figure 3). In the DCD-CN group, LDP was increased by an elevated left-ventricular volume.

**Figure 4:**
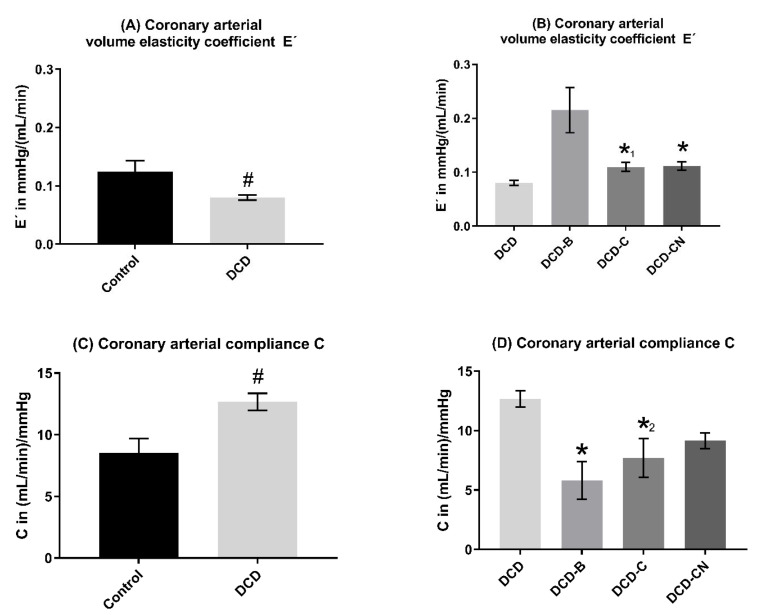
Coronary elasticity and compliance. ^#^ *p* < 0.05 compared to Control. * *p* < 0.05, *^1^ *p* = 0.066, and *^2^ *p* = 0.052 compared to DCD. DCD: Donation after circulatory death. C: Custodiol. CN: Custodiol-N. B: Blood.

E′ was significantly decreased and C was significantly increased in the DCD group compared to the control group (Figure 4). Four hours of MP resulted in an increase in E’ and a decrease in C. In DCD-B, C decreased the most and E’ increased the most. In the DCD-CN group, C remained the highest among all perfusion groups.

### 2.3. Endothelial Injury

**Figure 5:**
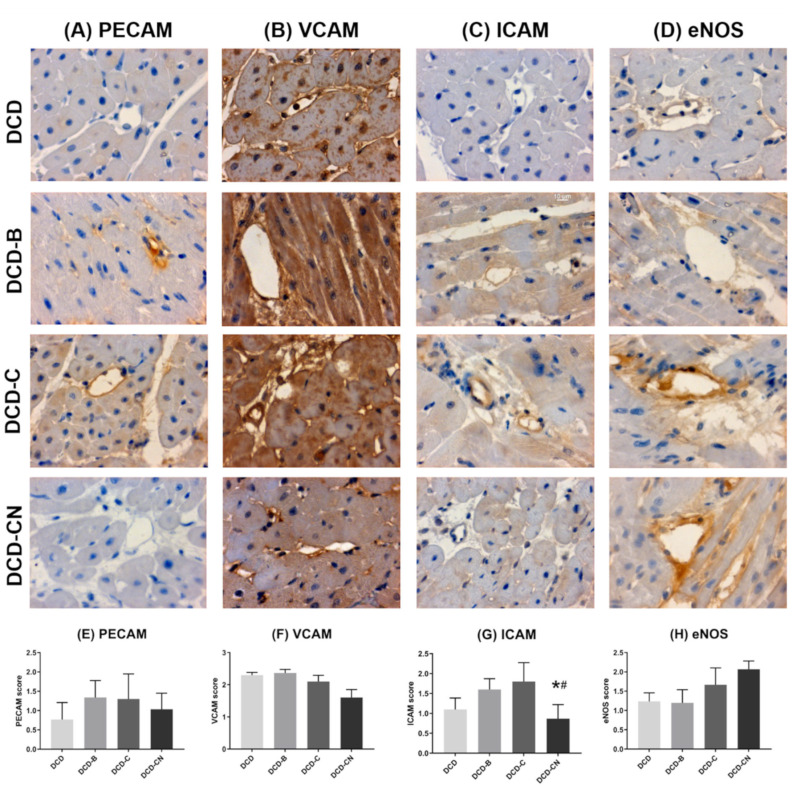
Immunoreactivity of cell adhesion molecules. (**A**–**D**) Representative photomicrographs are shown. (**E**–**H**) Semiquantitative scoring. B: Blood. C: Custodiol. CN: Custodiol-N. DCD: Donation after circulatory death. eNOS: Endothelial nitric oxide synthase. ICAM: Intracellular cell adhesion molecule-1. VCAM: Vascular cell adhesion molecule-1. PECAM: platelet endothelial cell adhesion molecule-1. * *p* < 0.05 vs. DCD. ^#^ *p* < 0.05 vs. DCD-B.

Blood perfusion resulted in an eNOS-score comparable to DCD (Figure 5). Custodiol-N showed the highest eNOS-score among all groups. ICAM and VCAM were majorly decreased in the DCD-CN group. Perfusion with blood and Custodiol resulted in an increased ICAM- and PECAM-expression, and an unchanged VCAM-expression compared with DCD. A decreased PECAM expression could be found in the DCD-CN group compared to blood or Custodiol perfusion. The missing significance of PECAM, VCAM, and eNOS results from the low sample size of N = 5 per group, considering the given standard error. However, the group size was powered for functional results.

### 2.4. Gene Expression

**Figure 6:**
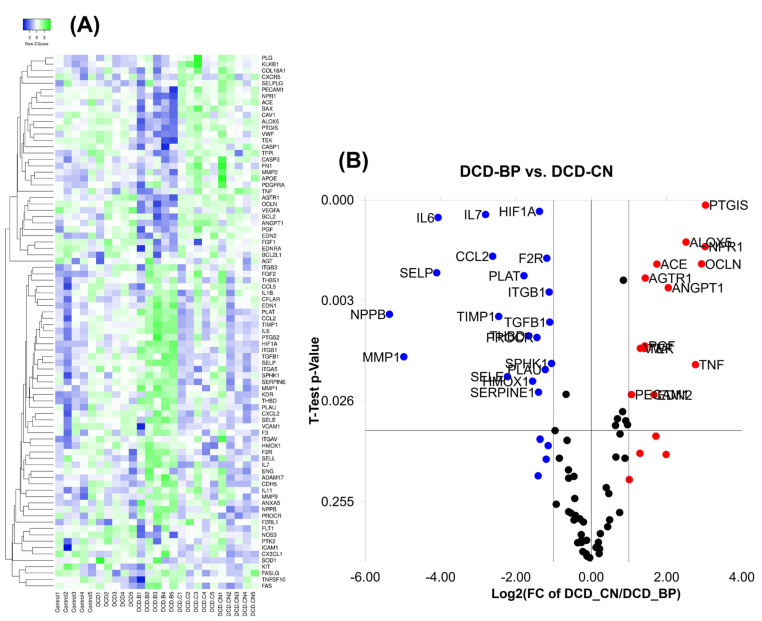
Gene expression. (**A**) For construction of the heatmap, a median linkage using Kendall’s tau was applied. (**B**) For the volcano plot comparison, a *t*-test with Bonferroni correction was applied. B: Blood. C: Custodiol. CN: Custodiol-N. DCD: Donation after circulatory death.

The direct expression comparison between DCD-BP and DCD-CN showed a downregulation of several genes, such as IL-6, IL-7, HIF1A, CCL2, MMP1, F2R, TIMP1, and NPPB, in DCD-CN (Figure 6). Other transcripts, such as PTGIS, ALOX5, OCLN, NPR1, AGTR1, ANGPT1, and TNF, were upregulated.

**Figure 7:**
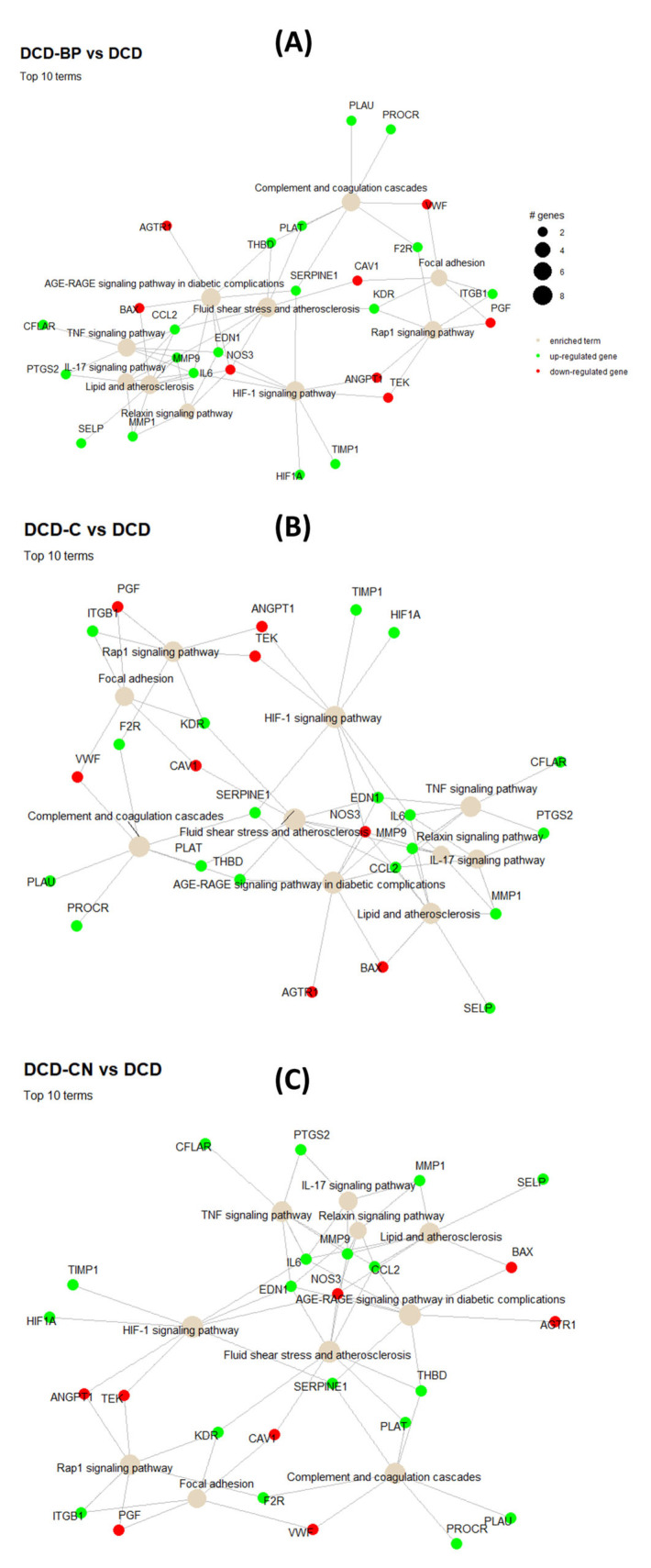
Pathway analysis of gene expression. For construction of the heatmap, median linkage using Kendall’s tau was applied. B: Blood. C: Custodiol. CN: Custodiol-N. DCD: Donation after circulatory death.

The DCD-CN group showed a downregulation of single genes, such as selectin E (SELE), selectin P (SELP), and interleukin(IL)-6, compared to the DCD group, while in DCD-B and DCD-C, these genes were upregulated (Figure 7). Furthermore, DCD-C showed an upregulation of the C-X-C Motif Chemokine Ligand 2 (CXCL2). Moreover, compared to DCD, the pathways involved in complement and coagulation cascades, focal adhesion, fluid shear stress, and the IL-6 and IL-17 pathways were upregulated only in DCD-B.

## 3. Discussion

We could demonstrate an improved developed pressure during systole, an improved slope of diastolic pressure decrement, an improved microvascular function, and less endothelial injury only after the MP of porcine DCD hearts with hypothermic, oxygenated Custodiol-N, but not with traditional Custodiol or normothermic blood. Furthermore, the pathways involved in complement and coagulation cascades, focal adhesion, fluid shear stress, and the IL-6 and IL-17 pathways were upregulated in DCD hearts only after blood perfusion.

### 3.1. Coronary Macro- and Microcirculation

The coronary circulation is adapted in an autoregulative manner to keep the myocardial perfusion constant under different CPP conditions, as first described by Bayliss et al. [10]. Depending on the current demands of the heart, the resistance vessels, mainly arterioles, contract or dilate to compensate for CPP variations and thereby regulate the flow, which actually reaches cardiomyocytes or immediately passes to the venous side by arterio-venous anastomoses.

The present study confirms that the perfusion solution has a major impact on vascular elastance, compliance, and coronary microvascular function. The mean overall compliance of the coronary vasculature was the worst in the blood perfusion group and was normalized to control levels in the Custodiol-N group. Moreover, only after Custodiol-N perfusion did a rising CPP lead to improved microvascular flow. After blood perfusion, a rise in CPP did not improve the microvascular flow and thereby seems to have passed arterio-venous anastomoses without improving the effective supply of cardiomyocytes. After perfusion with traditional Custodiol, a rise in CPP even in the low-pressure ranges improved microvascular flow. Nevertheless, the microvascular flow seemed uncoupled from CPP at pressure ranges above 40 mmHg in the DCD-C-, but not in the DCD-CN-group, which still constitutes an unphysiological state. Although the relationship between coronary flow and CPP was comparable, especially between DCD-C and –CN, the autoregulation of microvascular flow was only reconditioned by the novel Custodiol-N solution.

Myocardial microvascular flow can also be upregulated independently of perfusion variation by an increased LV-preload, as Kjekshus et al. demonstrated in a research project on dogs using labeled microspheres [11]. Next to the differences in CPP-dependent microvascular flow autoregulation, we could demonstrate that only Custodiol-N perfusion improves preload-dependent flow regulation.

### 3.2. Microvascular Dysfunction and Diastolic Performance

Diastolic function is classified as normal, impaired relaxation, pseudonormal, restrictive, or indeterminate. In the present study, we could show a slow, prolonged, and therefore impaired relaxation in all DCD groups, except after Custodiol-N perfusion. An impaired relaxation is apparent at an early stage of LV dysfunction. An impaired microvascular function induces a myocardial supply-demand mismatch by inadequate subendocardial perfusion. This, in turn, leads to decreased ATP production, resulting in diastolic cross-bridge cycling and, finally, impaired diastolic relaxation [12].

Nevertheless, the underlying pathomechanism that drives diastolic dysfunction as a result of CMD, as described by Daud et al., at a late stage after heart transplantation, might be different from an early coexistence of CMD and diastolic dysfunction [13]. Next to impaired active relaxation, increased myocardial stiffness can also be a potential reason for decreased diastolic function. To prove the effect of Custodiol-N perfusion on the prevention of myocardial stiffness in DCD hearts, studies on an ejecting heart would need to be performed after orthotopic transplantation in the future. Furthermore, it is known that an acidic condition of the initial reperfusion solution of DCD hearts is cardioprotective [14]. Therefore, perfusion with Custodiol-N, which has the most acidic pH value of 7.0, might show superior results compared to perfusion with Custodiol (pH 7.2) or blood (pH 7.35 to 7.45).

### 3.3. Molecular Mechanisms

Various mechanisms of PECAM-1 were discovered in the last decades. PECAM-1 is involved in many pathophysiological mechanisms of I/R injury, such as increasing endothelial permeability [15]. Considering the increased PECAM-1 expression in the DCD-B group, this could be a possible explanation for the increased edema formation in porcine DCD hearts after blood perfusion in a previous study [8]. Edema formation induces the extramural compression of microvessels, reducing microvascular flow [4]. This is predominantly a mechanical effect that could have contributed additionally to reduced microcirculation.

The production of nitric oxide (NO) by eNOS is an endothelial-dependent dilatory mechanism in the coronary microvasculature. NO is released from endothelial cells in response to various physiological stimuli [16]. The filling of the left ventricle was shown to directly impact NO release [17]. Therefore, it is reasonable that a stepwise increase in the left-ventricular filling induced an increased myocardial LDP. This physiological phenomenon was not visible in other groups. It is known that PECAM-1 knockout endothelial cells are characterized by increased eNOS activity and nitric oxide production [18]. Considering this, we conclude that the increased eNOS expression in DCD-CN hearts is at least partially dependent on reduced PECAM-1 expression. Another potential reason for improved microvascular function in the DCD-CN group could be that the Custodiol-N solution contains the amino acid L-arginine, which is an NO donor [19]. The continuous perfusion of the heart with Custodiol-N solution for 4 h potentially enables a saturation of cells with L-arginine, which also lasts during reperfusion with blood. It has also been shown in cell culture experiments with human aortic endothelial cells that an increased presence of L-arginine reduced the expression of ICAM [19], which is consistent with our results from the immunohistochemical staining of coronary arterioles (Figure 6).

Gene expression profiling and respective statistical analysis revealed differences in the up- and downregulation of single genes and multiple pathways that might cause the different impacts of the perfusion solutions on DCD hearts. SELE promotes endothelial leukocyte adhesion and is expressed after the cytokine stimulation of endothelial cells [20]. SELP mediates the interaction of activated endothelial cells with leukocytes [21]. Both were downregulated only after perfusion with Custodiol-N, which could be a potential reason for the improved microvascular function in the DCD-CN group. CXCL2 is produced by activated monocytes and neutrophils and was upregulated in the DCD-C group, suggesting a stronger activation of immune cells during reperfusion after machine perfusion with Custodiol [22]. IL-6 and -17 are pro-inflammatory cytokines that potentially increase the inflammatory reaction [23]. The upregulation of complement and coagulation cascades could be a potential reason for impaired microvascular flow in DCD-B. Furthermore, the upregulation of focal adhesion promotes the involvement of leukocytes in inflammatory processes in the coronary vascular endothelium. Considering the involvement of inflammatory pathways in DCD-B, we suggest verifying the efficacy of a cytokine adsorption device during the blood perfusion of DCD hearts or reperfusion after DCD heart transplantation. Activated leukocytes might also have an effect on the coronary endothelium. Therefore, we additionally suggest investigating the effect of a leukocyte filter after blood drainage before the priming of the perfusion system for the blood perfusion of DCD hearts.

IL-6, IL-7, and MMP1 increase I/R injury, which is associated with myocardial functional decline [24,25,26]. CCL2 expression is commonly increased after myocardial ischemia [27]. Consequently, a decreased CCL2 expression in DCD-CN highlights the reduced myocardial injury in this group. Interestingly, TIMP1 levels are increased in patients with diastolic dysfunction [28], which is consistent with the increased TIMP1 expression in the group with diastolic dysfunction (the DCD-B group) in the present work. NPPB expression increases in the myocardial infarction border zone and is important for functional recovery after infarction [29]; hence, in the DCD-BP group, a decreased infarction-related stadium was evident.

The reduced levels of OCLN in DCD-BP are consistent with results from Niu et al., who show a decreased ilium OCLN expression after cardiopulmonary resuscitation [30]. It was also shown that AGTR1 is downregulated in the infarcted myocardium in rats, which underlines the apparent myocardial injury in the DCD-BP group [31]. ANGPT1, which was upregulated in DCD-CN, promotes cardiac myocyte survival and limits ischemia-induced injury, which might explain the superior ventricular function in the DCD-CN group [32].

## 4. Materials and Methods

### 4.1. Animals and Anesthesia

The investigations were reviewed and approved (35-9185.81/G-150/19) by the appropriate institutional Ethical Committee for Animal Experimentation. Animals received humane care [33]. Animals were treated as described previously [8]. In brief, we sedated healthy pigs with a body weight of 40–45 kg with an intramuscular injection of ketamine (22.5 mg/kg; Bremer Pharma, Warburg, Germany) and midazolam (0.375 mg/kg; Hameln pharma plus, Hameln, Germany). Anesthesia was maintained intravenously with pentobarbital-sodium (15 mg/kg/h; Boehringer Ingelheim Vetmedicia, Ingelheim, Germany) through the ear vein. We used Dipidolor (1.125 mg/kg/h; Piramal Critical Care, Voorschoten, The Netherlands) for analgesia. We adjusted ventilation to maintain a partial oxygen (PaO_2_) pressure of around 200 mmHg and partial carbon dioxide (PaCO_2_) pressure of 35–45 mmHg. We established arterial vascular access in the femoral artery for monitoring arterial blood pressure and blood sampling.

### 4.2. DCD Model

The chest was opened by median sternotomy followed by pericardiotomy to expose the heart. To achieve systemic anticoagulation, we injected heparin (LEO Pharma, Neuisenburg, Germany) intravenously. According to a previously published model, DCD was induced by the termination of mechanical ventilation [34]. To allow maximal standardization, we applied a total warm ischemic period of 30 min, starting with the termination of mechanical ventilation. During this 30-min period, circulatory death occurred, and 5 min after circulatory arrest, we drained the blood from the donor animal to prime the perfusion system. Independent of when circulatory death occurred during this 30 min, the hearts were flushed with 2 L of 4 °C cold Histidine-Tryptophane-Ketoglutarate solution (Köhler Chemie GmbH, Bensheim, Germany), which is used routinely by our institution in clinical heart transplantation, after 30 min, and then procured.

### 4.3. Study Groups and Machine Perfusion

After cardioplegia flush, we connected the hearts to the perfusion system by a cannula in the ascending aorta. In terms of maintenance perfusion, the hearts were perfused either with warm (37 °C) oxygenated blood (DCD-B group, N = 5) or cold (4 °C) traditional Custodiol (DCD-C group, N = 5) or novel Custodiol-N (DCD-CN group, N = 5) solution. The composition of Custodiol and Custodiol-N is shown in Table 1.

**Figure 8:**
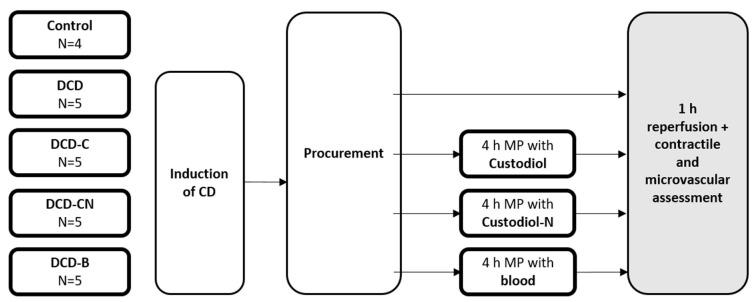
Study Groups. CD: Circulatory Death. DCD: Donation after circulatory death. C: Custodiol. CN: Custodiol-N. B: Blood.

After 4 h of maintenance perfusion, the hearts of all groups were reperfused with fresh blood for 60 min to mimic transplantation. Also, in the DCD-B group, fresh blood was used for reperfusion and functional analysis. During reperfusion, contractile and vascular function were assessed. In a fourth group (DCD group, N = 5), DCD hearts underwent immediate functional assessment without prior maintenance perfusion. In another group, which served as a control, no circulatory death was induced (Figure 8).

During four hours of maintenance perfusion in the DCD-C and DCD-CN groups, the crystalloid preservations solutions were oxygenated to a PaO_2_ of 180–200 mmHg, and the coronary perfusion pressure (CPP) was adjusted to 20 mmHg. During four hours of maintenance perfusion in the DCD-B group and the final functional assessment in all groups, heparin (5000 iU), sodium-chloride, magnesium-chloride, glucose, sodium-prednisolone, sodium hydrogen carbonate, and mannitol were added to the blood. Additionally, perfusion parameters were adjusted as follows: PaO_2_ of 180–200 mmHg, PaCO_2_ of 35–45 mmHg, pH of 7.35–7.45, and a perfusion pressure of 50–60 mmHg. Arterial and venous perfusate samples were measured every 30 min with a point-of-care analyzer (RAPID Point 500, Siemens, Erlangen, Germany) in all groups during four hours of maintenance perfusion and functional assessment.

### 4.4. Contractile and Vascular Functional Assessment

During reperfusion, contractility was measured with a left-ventricular balloon catheter inserted into the left ventricle through the mitral valve. The balloon was loaded with different volumes of 5, 10, 15, and 20 mL at a constant CPP of 60 mmHg. We also assessed the autoregulative function of the coronary microvasculature. Therefore, myocardial microcirculation was measured at different CPPs of 20, 40, 60, 80, and 100 mmHg with a constant left-ventricular preload volume of 10 mL. Myocardial microcirculation was measured by Laser-Doppler-Technology, as described in detail previously [8]. According to the technology, myocardial microcirculation is proportional to the Laser-Doppler-Perfusion (LDP) signal. In brief, a Laser-Doppler-Needle probe was inserted into the left ventricular anterior wall and was fixated with a 5/0 Prolene suture. Due to the dimensionless properties of LDP, the signal needs to be related to a baseline measurement, finally leading to relative LDP (RLDP) or fold change (FC) of LDP. A baseline measurement was performed at 20 mL CPP and 0 mL left-ventricular volume. We calculated the coronary arterial volume elasticity coefficient E’ and coronary arterial compliance C from the mean change of coronary flow by the stepwise elevation of PP by 20 mmHg.

### 4.5. Immunohistochemical Staining of eNOS, PECAM, ICAM, VCAM

After the microvascular functional assessment, samples of LV myocardial tissue were frozen in liquid nitrogen and stored at −80 °C. Moreover, additional tissue samples were conserved in paraformaldehyde solution and embedded in paraffin to be cut into 4 µm thick slices, which were placed on adhesive slides. The immunohistochemical staining of platelet endothelial cell adhesion molecule-1 (PECAM-1; 1:1000; Santa Cruz Biotechnology, Heidelberg, Germany), vascular cell adhesion molecule-1 (VCAM-1; 1:1000; Santa Cruz Biotechnology, Heidelberg, Germany), intracellular cell adhesion molecule-1 (ICAM-1; 1:100; Novusbio, Wiesbaden, Germany), and endothelial nitric oxide synthase (eNOS; 1:100; Cell signaling technology, Danvers, Massachusetts, USA) were performed according to the manufacturer’s instructions. For all described staining methods, the arterioles from six standardized areas per slice were analyzed using a semiquantitative scoring system, which assigns a scoring value of 0, 1, 2, or 3, depending on the intensity of the staining. The mean value per slice was calculated for further statistical analysis.

### 4.6. Gene Expression Analysis

The expression of 84 genes was analyzed using the RT^2^ PCR array (Qiagen, Hilden, Germany), as described elsewhere [8]. In brief, RNA was isolated from frozen samples of LV tissue of the anterior wall using the RNeasy Mini Kit (Qiagen, Hilden, Germany), followed by real-time quantitative reverse transcription PCR using an SYBR Green containing master mix (Qiagen, Hilden, Germany). RNA was converted into cDNA by the RT2 First Strand Kit (Qiagen, Hilden, Germany), and samples were transferred into a plate with pre-dispensed gene-specific primer sets. Housekeeping genes for beta-2-microglobulin, glyceraldehyde-3-phosphate dehydrogenase, hypoxanthine phosphoribosyl transferase 1, lactate dehydrogenase A, phosphoglycerate kinase 1, and ribosomal protein L13a were used for the normalization of gene expression values.

### 4.7. Statistical Analysis and Machine Learning Algorithm

Statistical analysis was performed using IBM SPSS Statistics for Windows (Version 25.0, IBM Corp. Armonk, NY, USA). The results are expressed as mean ± standard error. Homogeneity of variances was tested by the Levene’s test. Data were analyzed using a one-way analysis of variance (ANOVA) for multiple comparisons with the Tukey-adjustment of *p*-values in case of variance homogeneity and Games-Howell adjustment in case of variance inhomogeneity. A value of *p* < 0.05 was considered statistically significant.

The pathfindR package was used to identify significantly enriched pathways and generate comparison-specific heatmaps and network diagrams for gene expression. The comparisons DCD-CN vs. DCD, DCD-B vs. DCD, and DCD-C vs. DCD were analyzed to identify the impact of the specific perfusion solution on gene expression and pathway activation in DCD hearts. Differentially expressed genes (*p* < 0.1) were identified for these comparisons. The genes and *p*-values (Benjamini Hochberg adjusted) were mapped onto the STRING PIN (protein interaction network), which relates protein relationships to genes. An active subnetwork search using the greedy search algorithm was conducted, and the subnetworks were filtered based on a score metric calculated by the package. The top subnetworks were mapped onto KEGG genetic pathways. The enrichment scores for each pathway were calculated via agglomerated Z scores and used for heatmap construction. Identified subnetworks were used to draw the network diagrams.

## 5. Conclusions

Systolic function is of central importance in maintaining circulation in the body. Nevertheless, diastolic and vascular, especially microvascular function, are important for long-term outcomes after heart transplantation and should also be considered for the reperfusion, resuscitation, or reconditioning of DCD hearts.

Unlike the effect on systolic function, only perfusion with hypothermic, oxygenated Custodiol-N solution prevented porcine DCD hearts from early diastolic and coronary microvascular dysfunction, microvascular endothelial I/R injury, and the upregulation of pro-inflammatory pathways and complement cascades.

## Figures and Tables

**Table 1:** Composition of Custodiol and Custodiol-N.

	Custodiol	Custodiol-N
**Electrolytes (mmol/L)**		
Na^+^	16	16
K^+^	10	10
Mg^2+^	4	8
Ca^2+^	0.015	0.020
Cl^−^	50	30
**Buffer substances (mmol/L)**		
Histidine	198	124
Nα-acetyl-L-histidine	-	57
**Further aminoacids (mmol/L)**		
Tryptophan	2	2
a-Ketoglutarate	1	2
Aspartate	-	5
Arginine	-	3
Alanine	-	5
Glycine	-	10
**Oncotic agents (mmol/L)**		
Mannitol	30.0	-
**Sugars (mmol/L)**		
Sucrose	-	33
**Iron chelators (mmol/L)**		
Deferoxamine	-	0.025
LK-614	-	0.0075
	Blood perfusate	
Na^+^	135–145	
K^+^	4.0–5.5	
Ca^2+^	0.9–1.2	
Cl^−^	100–115	
Haematokrite	Approx. 20%	

## Data Availability

Data available on request.

## Abbreviations

ANOVA	Analysis of variance
CPP	Coronary perfusion pressure, Coronary perfusion pressure
CXCL2	C-X-C Motif Chemokine Ligand 2
DCD	Donation from circulatory death
eNOS	Endothelial nitric oxide synthase
FC	Fold change
HTK	Histidine-tryptophane-ketoglutarate
I/R	Ischemia/reperfusion
ICAM	Intracellular cell adhesion molecule-1
IL	Interleukine
LDP	Laser-Doppler-Perfusion
MP	Machine perfusion
MVD	Coronary microvascular dysfunction
PECAM-1	Platelet endothelial cell adhesion molecule-1
RLDP	Relative LDP
SELE	Selectin E
SELP	Selectin P
VCAM	Vascular cell adhesion molecule-1
